# Return to play following clavicular fracture – A systematic review and meta analysis

**DOI:** 10.1016/j.xrrt.2024.11.002

**Published:** 2024-12-14

**Authors:** Conor J. Kilkenny, Gordon R. Daly, Sean P. Whelehan, Danilo Vukanic, Maen Alrawashdeh, Fiona Boland, John F. Quinlan, Diarmuid C. Molony

**Affiliations:** a Department of Orthopaedics, Tallaght University Hospital, Dublin, Ireland; b Royal College of Surgeons in Ireland, Dublin, Ireland; c Faculty of Education & Health Services, University of Limerick School of Medicine, Castletroy, Co. Limerick, Ireland; d Data Science Centre, RCSI University of Medicine and Health Sciences, Dublin, Ireland

**Keywords:** Injury, Return to play, Sport, Athlete, Clavicle, Fracture

## Abstract

**Background:**

Clavicular fractures are common injuries in athletes, constituting up to 10% of all sport-related fractures. The location and severity of these fractures influence treatment decisions, which can range from conservative to operative management. Concerns exist regarding complications and delayed return to play (RTP), particularly for displaced midshaft and lateral fractures. Despite numerous studies on RTP following clavicle fractures, there is a lack of recent systematic reviews presenting comprehensive data on RTP rates and influencing factors. This systematic review aims to provide an overview of RTP in athletes following clavicle fractures, including an examination of fracture type, location, and management strategies.

**Methods:**

This systematic review, following Preferred Reporting Items for Systematic Reviews and Meta-Analyses guidelines, identified 33 clinical studies through searches in PubMed, EMBASE, Cochrane, CINAHL, Web of Science, and Scopus databases. Two independent reviewers conducted study selection, data extraction, and quality assessment, with discrepancies resolved by a third reviewer. Studies reporting on RTP after clavicular fractures, published in English, were included.

**Results:**

The review included studies involving a total of 1087 patients, reflecting a range of fracture characteristics and patient demographics. Overall, the RTP rate was 91%, with 86% of athletes returning to the same level of play. Rates varied based on the fracture location, with medial fractures showing the highest RTP (100%) and lateral fractures the lowest (78%). Operative and nonoperative management demonstrated similar RTP rates (92% vs. 91%), but operatively managed patients had higher rates of RTP to the preinjury level (92% vs. 78%). The mean time to RTP was 3.1 months for operatively managed fractures and 3.9 months for those managed nonoperatively.

**Conclusion:**

High rates of RTP are seen for athletes managed both operatively and nonoperatively following a clavicular fracture. Effective management of lateral clavicular fractures remains an ongoing challenge. Patients with high functional demands need careful consideration to optimise RTP outcomes. While operative management may offer superior RTP to the preinjury level, the decision should consider potential complications and patient preferences. Standardized reporting of RTP outcomes is essential for future research to facilitate comparison and optimize management strategies.

Clavicular fractures are a common pathology among athletes, accounting for up to 10% of all sport-related fractures.^1^^,^^6^ Allman classified clavicle fractures into groups I (midshaft), II (lateral), and III (medial).^24^^,^^28^ Thus, fractures are divided into those occurring in the clavicle's medial, middle, or lateral third. These injuries can present as undisplaced, displaced, or comminuted based on patterns. Clavicular fractures are most prevalent in American football, soccer, cycling, snowboarding, skiing, and wrestling.^30^ The fracture location and type influence subsequent management, which can be conservative or operative. In addition, the degree of displacement is an important consideration; radiographic parameters, such as 100% displacement and >2cm shortening, are indicators for the operative management of clavicular fractures.^12^^,^^22^ There is concern regarding potential complications of conservative management of displaced midshaft fractures resulting in decreased shoulder function and delayed return to play (RTP).^4^^,^^27^ Displaced lateral fractures are associated with high rates of nonunion and reduced shoulder function. Thus, operative management is considered for displaced midshaft fractures and lateral fractures.

RTP is a significant factor for athletes following shoulder surgery.^31^ Robertson et al found the overall RTP rate following midshaft clavicle fracture to be high.^25^ However, there is concern over reduced rates of return to the previous level of play and extended return times. Despite recent studies being available, no recent systematic reviews present up-to-date data on the RTP postclavicle fracture.

The authors aim to present an updated systematic review on RTP in athletes following clavicle fracture, encompassing fracture type, location, and management. This study hypothesised that RTP rates would be high following clavicle fracture; however, lateral clavicular fractures may have a reduced rate of RTP and extended RTP times.

## Methods

This systematic review was performed under the Preferred Reporting Items for Systematic Reviews and Meta-Analyses guidelines. All data included were from a previously published source. Two authors (C.J.K. and G.R.D.) formulated the study protocol.

### Search strategy

An electronic search was performed of the PubMed Medline, EMBASE, Cochrane, CINAHL, and Web of Science Scopus databases on 3 April 2024 for relevant studies for inclusion in this study. Two independent reviewers (C.J.K. and G.R.D.) performed the search using a predetermined search strategy. Search terms included the following: (‘clavicle’) AND (‘fracture’) AND (‘return’) AND (‘sport’). All study designs were included. Duplicates were manually removed. All titles and abstracts were initially independently screened by C.J.K. and G.R.D., followed by a full-text review of appropriate studies. In conflicts of opinion on inclusion between reviewers, a third author (S.P.W.) arbitrated on inclusion. The full search summary can be found within the Supplemental Figures.

### Eligibility criteria

The inclusion criteria were the following: (1) clinical study on clavicular fractures, (2) report on RTP, (3) published in a peer-reviewed journal, and (4) published in English.

The exclusion criteria were the following: (1) review studies, (2) case reports, (3) literature reviews and meta-analyses, (4) cadaveric studies, (5) biomechanical studies, (6) abstract only, and (7) studies reporting on return to activity but not RTP.

### Data extraction and quality assessment

Two independent reviewers (C.J.K. and G.R.D.) extracted the following data using a predefined electronic spreadsheet: (1) the first author, (2) year of publication, (3) study design, (4) number of athletes, (5) mean age of athletes, (6) level of exercise, (7) sex of athletes, (8) number of athletes with follow-up, (9) RTP rates in all athletes, collision athletes, overhead athletes, medial vs. midshaft vs. lateral fractures, and/or operative vs. nonoperative management. (10) RTP rates to same or higher level in all athletes, collision athletes, overhead athletes, medial vs. midshaft vs. lateral fracture, and operative vs. nonoperative management. (11) RTP timing in all athletes, collision athletes, overhead athletes, medial vs. midshaft vs. lateral fracture, and operative vs. nonoperative management. Level of evidence was assessed using guidelines from the Journal of Bone and Joint Surgery.^33^ Quality assessment was performed using the Newcastle-Ottawa score (NOS) by two independent reviewers, and^32^ in instances of disagreement, a third reviewer (S.P.W.) adjudicated.

### Statistical analysis

The total number of athletes, the number of athletes who returned to play, and the number of athletes who returned to play to the preinjury level was extracted for each study. Whether the fracture was managed operatively or nonoperatively and the fracture location (medial, mid, or lateral) was also recorded. Whether or not athletes were collision athletes was recorded. An athlete was considered to be a collision athlete if they participated in sports and activities that involve regular impact with opponents.^19^ Random-effect meta-analyses were conducted to estimate the pooled rate of RTP and RTP to the preinjury level for operatively managed and nonoperatively managed fractures, and for medial, mid, and lateral fractures. The *I*^2^ value was used to measure the overall variation due to heterogeneity across the different groups, <25% heterogeneity is considered low, 25 to 50% moderate, and >50% high.^11^ Statistical analyses were performed using Review Manager (RevMan) version 5.4 (Nordic Cochrane Centre, Copenhagen, Denmark) and Stata version 18 (Stata Statistical Software: Release 182023; StataCorp, College Station, TX, USA).

## Results

### Literature search

**Figure 1:**
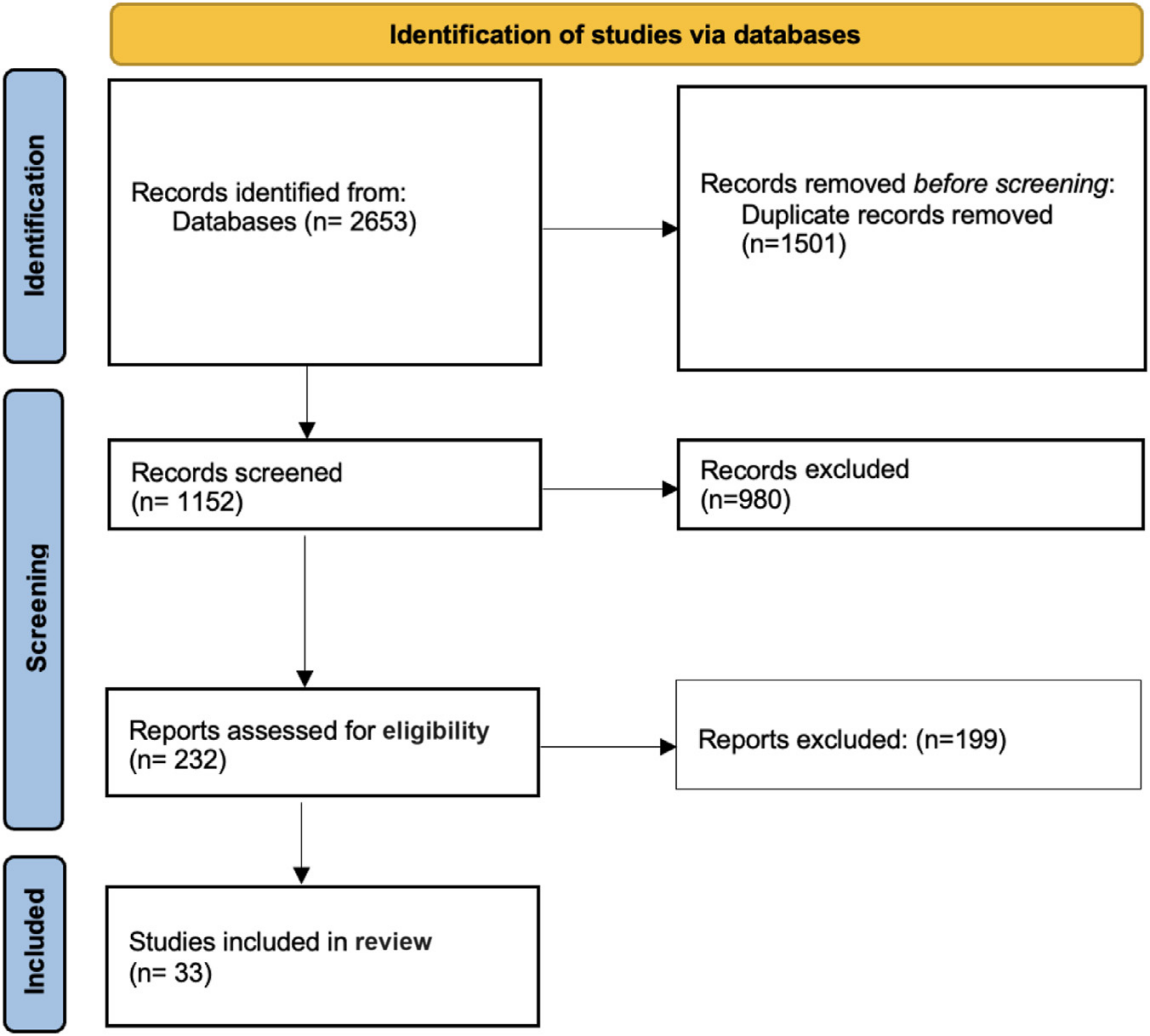
Preferred Reporting Items for Systematic Reviews and Meta-Analyses flow diagram. *From:* Page MJ, McKenzie JE, Bossuyt PM, Boutron I, Hoffmann TC, Mulrow CD, et al. The PRISMA 2020 statement: an updated guideline for reporting systematic reviews. BMJ 2021;372:n71. https://doi.org/10.1136/bmj.n71.

The initial literature search resulted in 2653 total studies. After the removal of duplicates, the articles were screened for inclusion and exclusion criteria, 1152 unique studies were evaluated, and full texts were assessed for eligibility. This review included 33 clinical studies (Fig. 1).

### Study characteristics and patient demographics

Our review found 33 studies, totaling 1087 patients, that met our inclusion criteria. The majority of the patients were male (83.7%), with a mean age of 28.36 years and a mean follow-up of 28.38 months. The mean NOS of the studies was 5.76. The study characteristics and patient demographics are seen in Table I.

**Table I:** Included study characteristics and patient demographics.

Lead author	Year	LOE	Newcastle-Ottawa score	N	Male	Female	Age (mean)	Follow-up (mo)
E. J. Osborn	2018	3	4	7	6	1	15.5	7
M. Ranalletta	2016	2	5	21	17	4	31.7	21
M. Ranalletta	2014	2	5	54	47	7	30.1	54
M. Ricks	2019	2	4	14	12	2	28.8	14
G. A. J. Robertson	2018	2	7	17	-	-	NR	17
Ahearn, B. M	2021	2	5	47	44	3	15.6	47
Carsen, S	2015	2	6	16	9	5	15.4	16
Davies, D	2009	2	6	56	44	12	38	56
Delvaque, J. G.	2019	2	6	19	18	1	37	19
Goldberg, J. A	1997	2	5	9	7	2	31	9
Goldfarb, C. A	2001	2	4	5	5	-	12.7	5
L. Robinson, R.	2015	2	4	22	17	5	14	22
K. Satyam,	2021	2	5	50	45	5	14.2	50
J. Schulz	2013	2	5	16	12	4	14.2	16
V. S. Sidhu	2015	2	5	27	26	1	37	24
N. A. S. M. Souza	2018	2	5	25	20	5	37	26
T. D. Tennent	2012	2	5	7	6	1	15.6	7
A. Titchener	2019	2	5	8	7	1	31.3	8
R. A. van der Linde	2022	2	7	274	-	-	45.9	274
O. Verborgt	2005	2	6	39	34	5	28	39
M. Wurm	2021	2	6	20	15	5	46.4	20
Hebert-Davies	2018	2	7	15	15	0	NR	15
Jack, R. A.	2017	3	7	16	16	0	27.8	17
Jubel, A.	2003	2	6	12	10	2	24.8	12
Karuppaiah, K.	2022	2	6	19	17	2	34	19
Lädermann	2017	3	6	42	35	7	42	42
Laux, C. J.	2021	3	6	16	12	4	NR	16
Lee, Y. S.	2008	3	7	52	27	25	40.5	52
Loriaut, P.	2013	3	6	21	14	7	33	21
Grassi, F. A	2001	3	7	80	66	14	33.7	80
Meisterling	2013	3	6	29	27	2	19	30
Jack, A. J	2017	2	8	30	30	0	NR	30
Vora, D.	2019	3	8	17	17	0	NR	17

*NR*, Not Recorded; *LOE*, Level of Evidence; *N*, Number.

### Return to play

The overall rate of RTP was 91%, with 86% of athletes returning to the same level of play. The mean time to RTP was 3.35 months. Among collision athletes, the overall rate of RTP was 94%, with 92% returning to the same level of play. The mean time of RTP in collision athletes was 3.47 months. In overhead-throwing athletes, the overall rate of RTP was 100%, with 100% returning to the same level of play. The mean time of RTP in overhead athletes was 2.3 months (Table II).

**Table II:** RTP outcomes.

Outcome	No. of studies	Result
Rate of RTP	33	91% (992/1087)
RTP at same or higher level (%)	30	86% (858/998)
RTP time (mean mo)	23	3.35
Rate of RTP for collision athletes	14	94% (202/216)
RTP at same or higher level for collision athletes (%)	14	92% (199/216)
RTP time for collision athletes (mean mo)	11	3.47
Rate of RTP for overhead athletes	5	100% (18/18)
RTP at same or higher level for overhead athletes (%)	5	100% (18/18)
RTP time for overhead athletes (mean mo)	4	2.3
Rate of RTP for operative management	28	92% (598/653)
RTP at same or higher level for operative management (%)	25	92% (538/587)
Timing of RTP for operative management (mean mo)	21	3.1
Rate of RTP for nonoperative management	9	91% (380/417)
RTP at same or higher level for nonoperative management (%)	7	78% (307/393)
Timing of RTP for nonoperative management (mean mo)	6	3.9

*RTP*, return to play.

The rate of RTP for patients who underwent operative management was 92%, with 92% of athletes returning to the same level of play. The mean time of RTP was 3.1 months. The rate of RTP for patients who underwent nonoperative management was 91%, with 78% of athletes returning to the same level of play. The mean time of RTP was 3.9 months (Table II).

**Figure 2:**
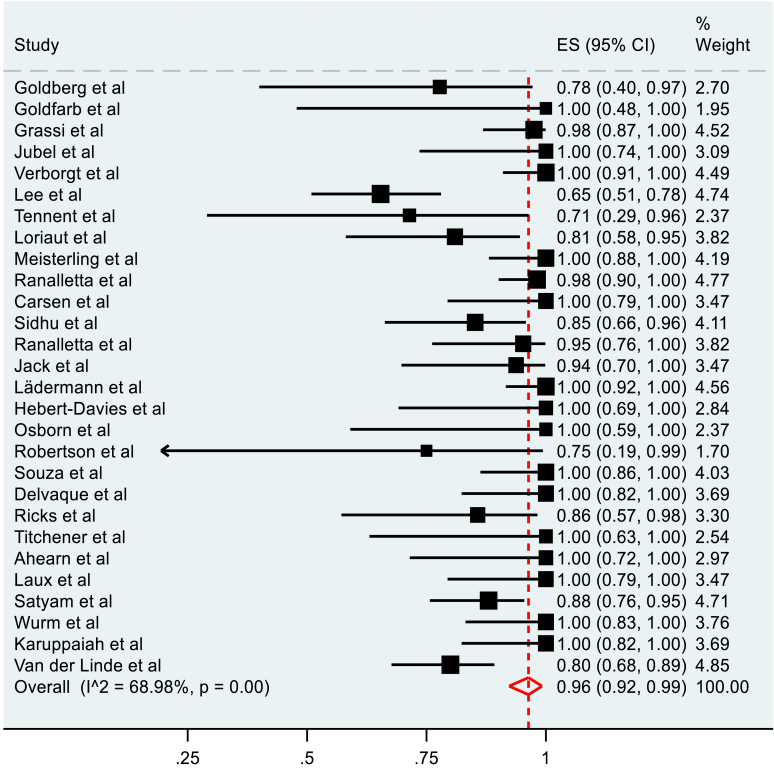
Pooled meta-analysis of overall RTP in operatively managed clavicle fractures. *RTP*, return to play.

**Figure 3:**
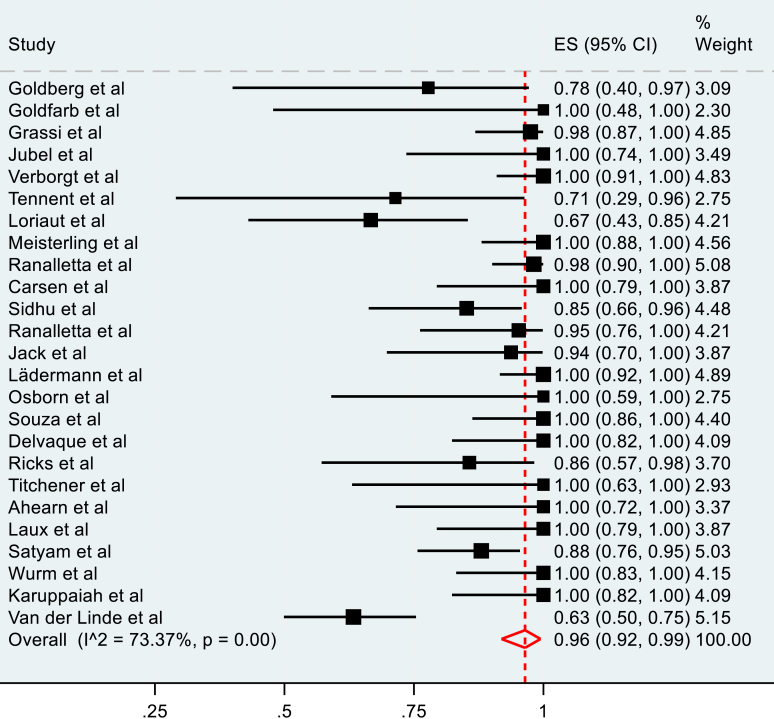
Pooled meta-analysis of RTP to preinjury level in operatively managed fractures. *RTP*, return to play.

The meta-analysis of the rate of RTP of operatively managed fractures included 28 studies. A pooled rate of 96% (95% C.I, 92-99%) was observed (Fig. 2). The pooled rate of RTP to the preinjury level for operatively managed fractures was 96% (95% C.I, 92-99%), as seen in Figure 3. Both observations had high heterogeneity, measured at 68.98% and 73.37%, respectively.

**Figure 4:**
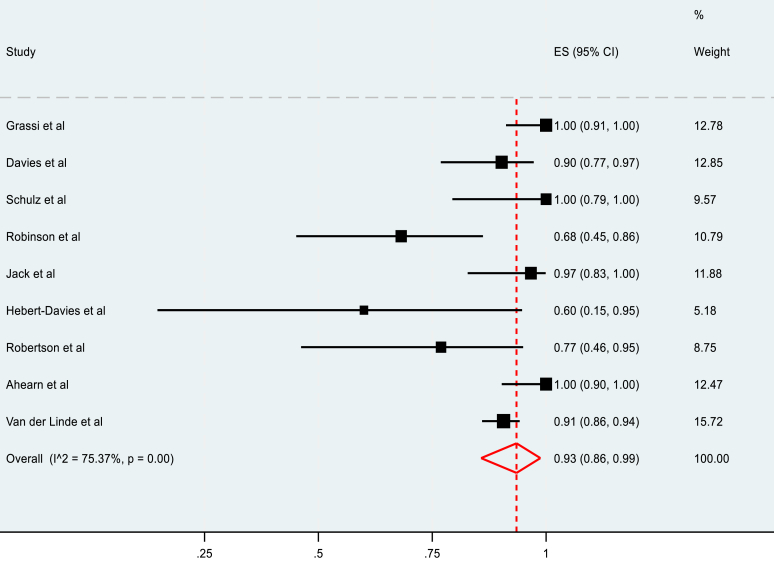
Pooled meta-analysis of rate of RTP in nonoperatively managed fractures. *RTP*, return to play.

**Figure 5:**
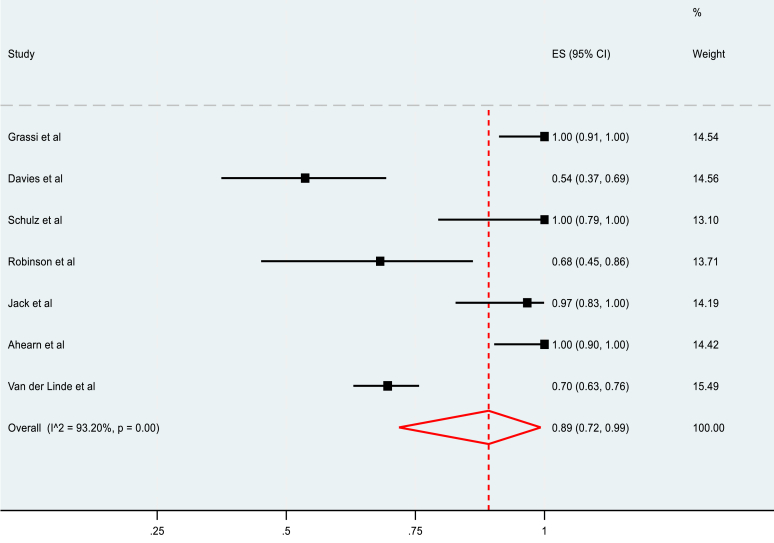
Pooled meta-analysis of rate of return to the preinjury level of play in nonoperatively managed fracture.

The meta-analysis of the rate of RTP in nonoperatively managed fractures included 9 studies and reported a pooled rate of 93% (95% C.I, 86-99%), as summarized in Figure 4. The rate of RTP to the preinjury level in nonoperatively managed fractures was 89% (95% C.I, 72-99%) in 7 included studies (Fig. 5). Heterogeneity was also high in both cases, measuring 75.37% and 93.20%, respectively.

**Figure 6:**
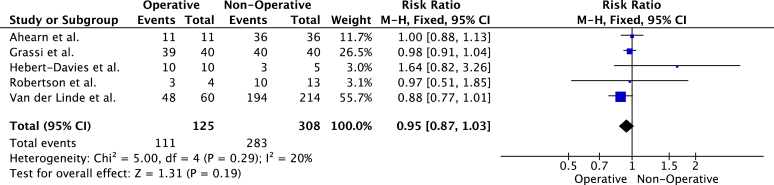
Meta-analysis of overall rate of RTP in studies comparing operative vs. nonoperative fracture management. *RTP*, return to play.

**Figure 7:**
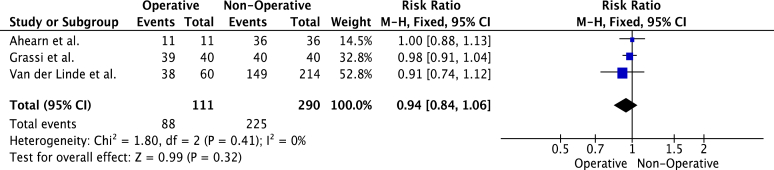
Meta-analysis of rate of RTP in to preinjury level in studies comparing operative vs. non-operative management. *RTP*, return to play.

Five studies compared RTP rates of operatively and nonoperatively managed fractures. Athletes with operatively managed fractures were less likely to RTP (Fig. 6). However, the differences were not statistically significant (risk- ratio, 0.95 (95% C.I, 0.87-1.03), *P* = .19). Heterogeneity was low (I^2^ = 20%, *P* = .29). Three studies reported on the level of RTP of operative vs. nonoperatively managed fractures. Similarly, operatively managed patients were less likely to RTP to the preinjury level (Fig. 7). However, the difference between the groups was not statistically significant (risk ratio, 0.94 (95% C.I, 0.84-1.06), *P* = .32).

For patients with medial clavicular fractures, the rate of RTP was 100%, with 90% returning to the same level of play. Among patients with mid-clavicular fractures, the rate of RTP was 91%, with 91% returning to the same level of play. The mean time of RTP in mid-clavicular fractures was 2.5 months. Patients with lateral clavicular fractures had rates to RTP of 78%, with 65% returning to the same level of play. The mean time of RTP in lateral clavicular fractures was 3.47 months (Table III).

**Table III:** Comparison of RTP by location of clavicular fracture.

Outcome	Medial	Mid	Lateral
Total	20	402	437
Rate of RTP	100% (20/20)	91% (367/402)	78% (341/437)
RTP at same or higher level (%)	90% (18/20)	91% (367/402)	65% (283/437)
Timing of RTP (mean mo)	N/A	2.5	3.47

*RTP*, return to play.

**Figure 8:**
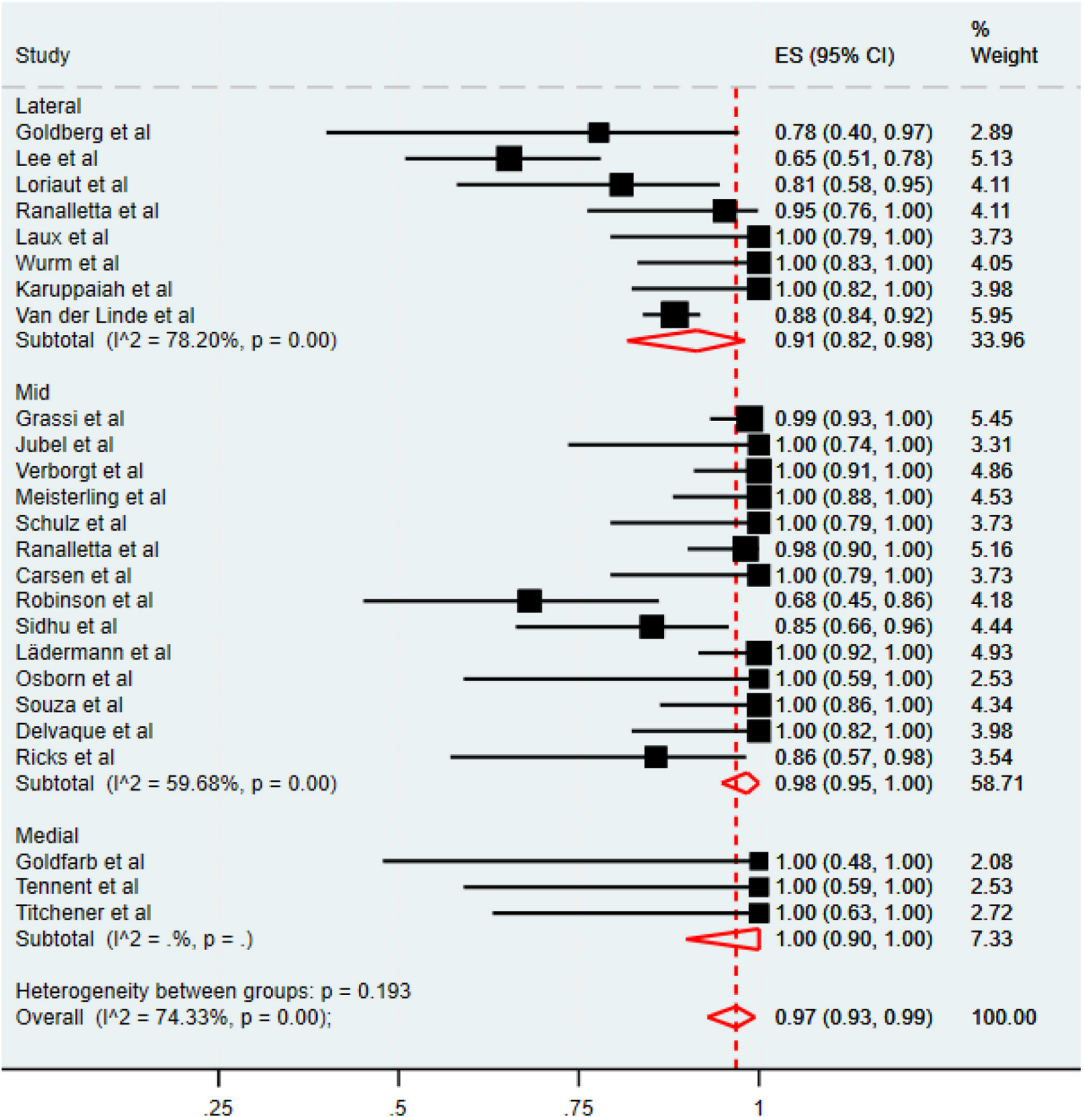
Pooled meta-analysis of rate of RTP in medial, mid, and lateral fractures. *RTP*, return to play.

The pooled RTP rate for all fracture locations was 97% (95% C.I. 0.93-99%), as seen in Figure 8. However, heterogeneity between all the groups was high, (I^2^ = 74.33%, *P* < .001). Lateral fractures had the lowest RTP of 91%, (95% C.I. 82%-98%); however, a large variability of outcomes in the 8 included studies was observed (I^2^ = 78.20%, *P* < .001). Middle-third fractures had a pooled return of 98% (95% C.I, 95%-100%), but similarly, the 14 studies included were highly heterogenous (I^2^ = 59.68%, *P* < .001). Only three studies reported RTP for medial fractures. All three reported an RTP rate of 100% (95% C.I, 90%-100%).

**Figure 9:**
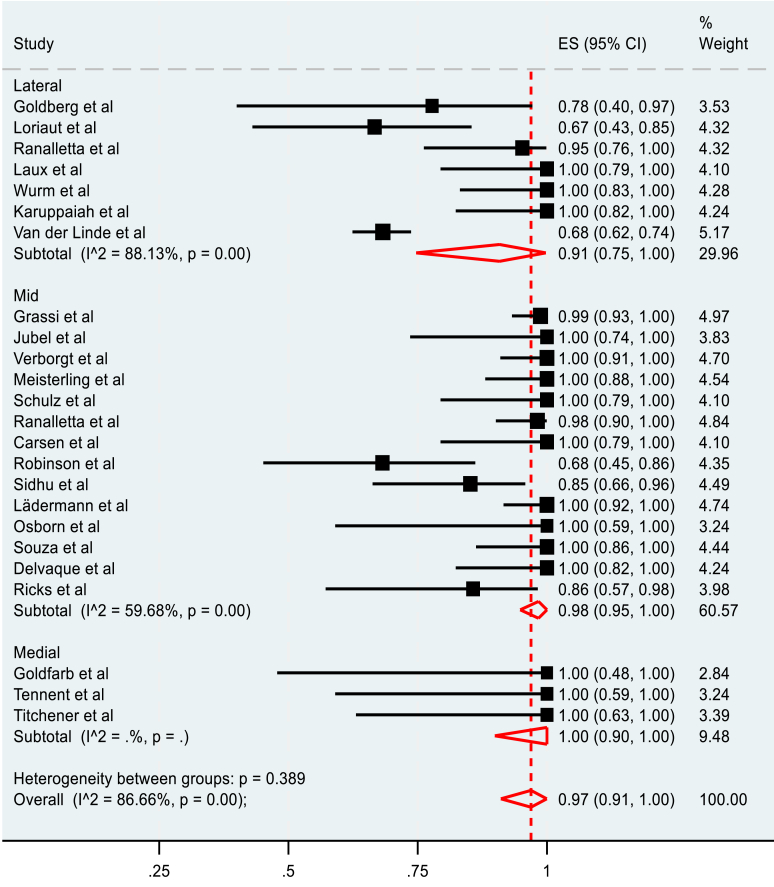
Pooled meta-analysis of rate RTP to the preinjury level for medial, mid, and lateral fractures. *RTP*, return to play.

The pooled meta-analysis of the rate of RTP to the pre-injury level is summarised in Figure 9. Similarly, the pooled rate of RTP to the preinjury level was 97% (95% C.I. 91%-100%). A high degree of heterogeneity was again observed between all the studies (I^2^ = 86.66%, *P* < .001). Athletes with lateral fractures were the least likely to return to the preinjury level of play, showing a pooled rate of 91% (95% C.I. 75%-100%). Middle clavicle fractures had a pooled rate of RTP to the preinjury level of 98% (95% C.I, 95%-100%). All patients in the three studies that reported the level of RTP for medial fractures reported an RTP of 100% to the preinjury level (95% C.I, 90%-100%). However, the heterogeneity was high in all cases.

## Discussion

This meta-analysis demonstrates a high rate of RTP following clavicular fractures. The RTP rates did not differ significantly between patients who were treated operatively versus nonoperatively; however, operatively managed patients appear to have quicker rates of RTP. Additionally, the highest rates of RTP were found among collision and overhead athletes. However, patients who suffered a lateral clavicular fracture had lower rates of RTP (78%), and even fewer returned to sport at the same level or above (65%), as demonstrated in Table III. This is significantly lower than in athletes with middle and medial clavicle fractures, indicating that these injuries have a substantial impact on future levels of activity and sport participation.

It is important to note that fracture severity and radiographic parameters affect management. Surgical intervention is indicated for completely displaced clavicular fractures, fractures shortened greater than 2 cm, or fractures that are significantly comminuted.^12^^,^^22^^,^^29^ Therefore operatively managed fractures may be inherently more displaced and represent a higher-level injury, which could also affect RTP. However, the findings of this study suggest that while operatively managed patients have similar rates of RTP to patients managed nonoperatively, they are more likely to return to a higher level.

While avoiding the risks of surgery may be of benefit to many patients, the requirement for a quick RTP for higher-level athletes may be more likely to opt for operative management.^9^ Operatively managed patients may be more likely to be adherent to their rehabilitation protocol, which is likely a contributing factor to operatively managed patients returning to a higher level. It is also important to consider other factors, which would contribute to a patient being managed operatively or nonoperatively. Factors, such as time of season and age, could also affect decision-making around management strategy. Furthermore, exposing patients to surgery carries the potential for certain complications, including infection, nonunion or delayed union, nerve palsy, and prominent metalwork.^20^ It is crucial that athletes are aware of these potential risks when deciding whether or not to undergo surgical management. The operative techniques used to manage clavicular fractures remain controversial. The most common surgical procedure would be open reduction and internal fixation, but other methods, such as intramedullary pins, nails, or wires, which allow a minimally invasive approach, are also utilised.^23^ Future studies should also focus on assessing outcomes based on surgical techniques to help guide future practice.

There is a paucity of studies assessing RTP based on the location of clavicle fractures. Likewise, very few studies have compared RTP outcomes directly comparing operative vs.non-operative treatment methods. The overall rate of RTP was high following clavicular fracture. Only one previous systematic review has focused on RTP outcomes following clavicular fracture.^25^ Robertson et al found a 92% rate of RTP, which is similar to the findings in our study.^25^

Systematic reviews reporting on injuries of different anatomical regions in shoulder surgery have found high rates of RTP, such as rotator cuff repair (85%),^16^ acromioclavicular joint dislocation (91.5%),^5^ arthroscopic Bankart (81%),^18^ and Latarjet procedures (89%).^13^ However, these studies do not mention the specific reasons why athletes do not RTP. The reasons for this are probably multifaceted and difficult to quantify. Hurley et al previously noted that other factors, apart from the physical limitations, can hinder an athlete from a full return to sport.^13^ These include changes in lifestyle (such as deciding to stop participating in nonprofessional sports or reducing the level of competition) or a fear of injury despite being physically capable. Similarly, the timing of RTP can be influenced by the time of season, with athletes more likely to take a prolonged rehabilitation period during the off season.^13^ These factors may significantly influence the reported rates of return to sports. In future research, it would be valuable to further evaluate the factors contributing to the decision not to return to the preinjury level of sports.

This study shows that rates and timing of RTP vary based on fracture location, with lateral clavicular fractures being associated with inferior RTP outcomes. Management of lateral clavicular fractures in the athletic population is debated, particularly in patients with displaced fractures. The decreased RTP may be attributable to the findings that displaced lateral clavicle fractures are associated with a higher incidence of nonunion and cosmetic deformities.^26^^,^^21^ Compared to Robertsons' systematic review, rates of RTP following lateral clavicular fracture were as high as 86%, with 78% returning to the same level.^25^ Thus, this study found the rate of RTP following lateral clavicular fracture to be lower than previously reported. Karuppaiah et al’s study included in this review assessed outcomes for patients following a lateral end-locking plate augmented with a coracoid anchor. This study showed that it was a reliable fixation method and avoided the increased complications that are associated with the use of a hook plate, such as dislocation of the hook, fracture medial to the plate, nonunion, AC joint arthrosis, acromion fracture, and acromion osteolysis.^8^^,^^10^^,^^15^ Focused studies are required to better elucidate the benefits of different surgical techniques and their impact on RTP.

It is thought that collision athletes are at an increased risk of suffering a clavicular fracture.^2^^,^^7^^,^^14^ Given that this cohort is at an increased risk of suffering from this pathology, the results of our study are encouraging. RTP rates for collision athletes are as high as 94%, and in studies where the level of RTP was reported, 92% returned to the same level or above (Table II). In one of the included studies, variable RTP outcomes were found for National Football League athletes dependent on position. Running backs and quarterbacks were found to have worse RTP outcomes. This was thought to be due to the effect of being tackled rather than being the tackler, where one can appropriately position their body and avoid contact with their shoulder.^14^

When assessing the RTP, it is worth noting that there was a significant variation in the outcomes reported within the individual studies. This limits our ability to draw definitive conclusions on a superior management strategy or inferior fracture locations regarding RTP. Also, only 8 of the included studies can be classified as having a “good” quality of evidence, according to the NOS criteria. This has also been noted in other systematic reviews reporting on RTP.^3^^,^^13^^,^^17^ Future researchers should be encouraged to publish a standardized set of RTP outcomes. This would allow for a more consistent comparison between studies, reducing the heterogeneityin systematic reviews. With only a few studies classified as having a high quality of evidence, more high-quality studies with standardized RTP measures are needed to achieve this goal.

### Limitations

As with all systematic reviews, this study possesses inherent limitations and biases, which stem from the included studies and their respective limitations. Due to constraints in the data reported in some of the included studies, our ability to analyse demographic factors as potential risk factors for inability to RTP was restricted. The authors note that there is a heterogeneous group of patients with different types of clavicular fractures, management strategies, and reported RTP outcomes included across studies. Dividing outcomes by location of fracture is important as these groups have demonstrated different levels of return to sport. A key limitation of this study is the lack of reported breakdown of RTP outcomes by specific sports, age, and degree of fracture displacement, limiting the capacity for subgroup analysis. Acknowledging these factors in future research could enhance the understanding of RTP dynamics following clavicular fractures. Additionally, only five studies reported on RTP in both groups and three on the level of RTP, severely limiting a comparative meta-analysis. The heterogeneity and lack of detail reported in the majority of studies limit our ability to draw higher-quality conclusions from this meta-analysis and highlight the need for further research on the topic.

## Conclusion

High RTP rates are seen for athletes managed both operatively and nonoperatively following a clavicular fractures. Effective management of lateral clavicular fractures remains an ongoing challenge, and RTP after these injuries is reduced compared to other locations. Patients with high functional demands need careful counselling to optimise RTP outcomes. While operative management may offer superior RTP to the preinjury level, the decision should consider potential complications and patient preferences. Standardised reporting of RTP outcomes is essential for future research to facilitate comparison and optimise management strategies.

## Disclaimers:

Funding: No funding was disclosed by the authors.

Conflicts of interest: The authors, their immediate families, and any research foundation with which they are affiliated have not received any financial payments or other benefits from any commercial entity related to the subject of this article.
