# Supplementary material for: Return to play following clavicular fracture – A systematic review and meta analysis
Source: JSES Rev Rep Tech. 2024 Dec 14;5(2):259–69. doi: 10.1016/j.xrrt.2024.11.002 (PMC12047575; doi:10.1016/j.xrrt.2024.11.002)
Supplement: Supplementary Figure 1 [file mmc1.pdf]

Supplementary Figures

Summary table of pooled meta analysis based on fracture location

Outcome	Medial	Mid	Lateral
RTP - ES (95% CI)	1.00	0.98	0.91
I^2	0%	59.68%	78.2%
RTP at same or higher level — ES (95% CI)	1.00	0.98	0.91
I^2	0%	59.68%	59.68%

Summary table of pooled meta analysis operative vs. nonoperative management

Outcome	Operative	Nonoperative
RTP - ES (95% CI)	0.96	0.93
I^2	68.98%	75.37%
RTP at same or higher level — ES (95% CI)	0.96	0.89
I^2	73.37%	93.2%

RTP, return to play; *ES*, estimates.

Full Search Strategy:

	PUBMED	3Apr24
1	Clavicle[Mesh] OR "clavicle"[Text Word] OR "clavicular"[Text Word]	11,829
2	“fracture*”[Text Word]	354,887
3	1 AND 2	4671
4	“athlet*"[Text Word] OR "sport*"[Text Word]	181,870
5	"return"[Text Word] OR "resume"[Text Word]	140,611
6	3 AND 4 AND 5	88
	EMBASE (Elsevier)	
1	‘Clavicle’/exp OR clavicle:ti,ab,de,kw OR clavicular:ti,ab,de,kw	17,143
2	fracture*:ti,ab,de,kw	484,234
3	1 AND 2	6997
4	athlet*:ti,ab,de,kw OR sport*:ti,ab,de,kw	235,821
5	return:ti,ab,de,kw OR resume:ti,ab,de,kw	199,896
6	3 AND 4 AND 5	148
7	LIMIT 6 2013-2024, English	107
	CINAHL (Ebscohost)	
1	(clavicle OR clavicular) AND (fracture*)	2659
2	athlet* OR sport*	148,733
3	return OR resume	167,234
4	1 AND 2 AND 3	158
5	LIMIT 4 to CINAHL only (excluding Medline); 2013-2024; English; Peer reviewed	45
	Cochrane Database of Systematic Reviews and Central Registry of Clinical Trials (Wiley)	
1	(clavicle OR clavicular) AND (fracture*):ti,ab,kw	370
2	(athlet* OR sport*):ti,ab,kw	20,652
3	(return OR resume):ti,ab,kw	27,846
4	1 AND 2 AND 3	
	Web Of Science, Science and Social Science Citation Indexes (Clarivate)	
1	TS ((clavicle OR clavicular) AND (fracture*)) AND (athlete* OR sport*) AND (return OR resume)	76
	Scopus (Elsevier)	
1	TI-AB-KY ((clavicle OR clavicular) AND (fracture*)) AND (athlete* OR sport*) AND (return OR resume)	130
2	LIMIT 1 to 2013-2024	91
